# The efficacy and safety of immune-checkpoint inhibitors plus chemotherapy versus chemotherapy for non-small cell lung cancer: An updated systematic review and meta-analysis

**DOI:** 10.1371/journal.pone.0276318

**Published:** 2024-02-06

**Authors:** Jing Kang, Jun Zhang, Zongsheng Tian, Ye Xu, Jiangbi Li, Mingxian Li

**Affiliations:** 1 First Hospital of Jilin University, Changchun, China; 2 Jilin Medical University, Jilin, China; 3 Laboratory of Tumor Targeted Therapy and Translational Medicine, Jilin, China; Roswell Park Cancer Institute, UNITED STATES

## Abstract

**Background:**

Immune-checkpoint inhibitors(ICIs) combined with chemotherapy are emerging as an effective first-line treatment in advanced non-small cell lung cancer (NSCLC); however, reports on the magnitude of effectiveness and safety are conflicting.

**Methods:**

Relevant articles published before February 2022 were searched in PubMed, Embase, and the Cochrane Library. The study included all randomized controlled trials that evaluated ICIs with chemotherapy versus chemotherapy for the treatment of NSCLC. Among the outcomes were overall survival (OS), progression-free survival (PFS), objective response rate (ORR), and treatment-related adverse events (TRAEs).

**Results:**

Our meta-analysis included a total of 12 studies. Overall analysis indicated that ICIs plus chemotherapy could significantly improve OS (HR = 0.79; 95% CI: 0.74–0.84; *I*^2^ = 44.4%, *P* = 0.055), PFS (HR = 0.62; 95% CI: 0.59–0.67; *I*^2^ = 75.3%, *P* = 0.000), and ORR (RR = 1.48; 95% CI: 1.27–1.73; *I*^2^ = 79.0%, *P* = 0.000) when compared to chemotherapy treatments. Subgroup analysis showed that PD-1/PD-L1 inhibitors combined with chemotherapy significantly improved OS, PFS, and ORR when compared with chemotherapy with decreased grade 1–2 TRAEs. In addition, female patients with nonsquamous histology might receive more OS benefit from ICIs plus chemotherapy when compared to chemotherapy alone. Despite the fact that CTLA-4 inhibitors combined with chemotherapy increased PFS, there were no benefits gained in OS nor ORR. When PD-L1/CTLA-4 inhibitors were added to chemotherapy, the risk of grade 3–5 adverse events increased whereas PD-1 inhibitors did not.

**Conclusions:**

ICIs plus chemotherapy, compared with chemotherapy, were associated with significantly improved PFS, ORR, and OS in NSCLC therapy. However, PD-L1/CTLA-4 inhibitors plus chemotherapy could increase the risk of grade 3–5 adverse events, but not PD-1 inhibitors plus chemotherapy.

## Introduction

Lung cancer is the primary cause of cancer-related deaths [1] with an estimated 2.20 million new cases and 1.79 million deaths every year, and 85% of all primary lung cancers are NSCLCs [2]. In recent decades, platinum-based chemotherapy has been widely used as the first-line treatment for advanced NSCLC [3]. However, chemotherapy has reached a plateau with only modest efficacy, and the 1-year survival of platinum-based chemotherapy for advanced NSCLC was only 34% [4]. Although some patients can obtain the desired effects with targeted medicines, treatment is limited to patients with epidermal growth factor receptor (EGFR) mutations, BRAF V600E mutations, ROS proto-oncogene receptor tyrosine kinase 1 (ROS1) rearrangements and anaplastic lymphoma kinase (ALK) rearrangements. Nevertheless, these mutations are found in only 30% of NSCLC patients [5]. There exists an urgent need for more effective and safer treatments for advanced NSCLC.

Significant progress has been achieved in the first- and second-line immunotherapy of advanced NSCLC in recent years, particularly immune-checkpoint inhibitors, (ICIs)including programmed death-1 (PD-1) inhibitors, programmed death-ligand 1 (PD-L1) inhibitors, and cytotoxic T lymphocyte associated antigen-4 (CTLA-4) inhibitors [6, 7]. ICIs function by releasing the inhibitory brakes of T cells resulting in immune system activation of anticancer [8]. Chemotherapeutic drugs may have immune-potentiating effects in some circumstances according to preclinical studies [9]. Increasing data indicate that combination ICIs are more effective than chemotherapy in patients with metastatic NSCLC as these patients exhibit considerably prolonged OS and PFS [10–14]. In addition, ICIs plus chemotherapy, which have a lower risk of immune-related adverse events than ICIs [15], are emerging as an effective first-line treatment for patients with advanced NSCLC [16]. However, reports on the magnitude of effectiveness and safety are conflicting.

There have been a handful of systematic reviews and meta-analyses focusing on the efficacy and safety of combination therapy with ICIs in NSCLC thus far [10, 14, 15, 17]. However, the results from these trials have not been consistent, which may result from variations in study design and insufficient sample size. Recently, an abundance of large RCTs have updated their recent trial data [13, 18–20]. Moreover, we are witnessing the emergence of new RCTs [21, 22], which will provide more comprehensive data for analysis. Thus, we aimed to conduct a systematic review and meta-analysis of randomized trials to assess the efficacy and safety of ICIs plus chemotherapy in patients with advanced NSCLC.

## Methods

The present systematic review conforms to the "Preferred Reporting Items for Systematic Reviews and Meta-Analyses" (PRISMA) statement (S1 Checklist) [23]. The registration number for systematic review is INPLASY202250156.

### Literature search strategy

Two independent reviewers (i.e., Jing Kang and Jun Zhang) conducted a systematic search of the PubMed and EMBASE databases as well as the Cochrane Library for relevant articles published before February 2022. We used the following text words as search terms: “carcinoma, non-small-cell lung” AND “immune therapy” OR “immune checkpoint blockade” OR “immune checkpoint inhibitor” OR “PD-1” OR “PD-L1” OR “CTLA-4” OR “durvalumab” OR “avelumab” OR “tremelimumab” OR “atezolizumab” OR “nivolumab” OR “pembrolizumab” OR “ipilimumab” OR “immune vaccine” AND “randomized controlled trial”. The detailed search strategies are presented in S1 Table. Additional studies were discovered by searching the references of relevant articles and reviews.

### Selection criteria

Studies were considered eligible if they met the following criteria: (1) they were randomized controlled trials published in English, (2) they were histologically confirmed to be advanced NSCLC patients, (3) they reported OS, PFS, ORR, and TRAEs, (4) the intervention group received ICIs plus chemotherapy while the control group received chemotherapy, and (5) when numerous papers reporting the same trial were found, the most current or most complete publications were chosen. The exclusion criteria were as follows: (1) duplicate articles, (2) reviews, meta-analyses, case reports, editorials, and letters, (3) molecular biology or animal research, and (4) retrospective or prospective observational cohort studies. The articles were screened separately by Jing Kang and Jun Zhang based on the title and abstract. The whole texts were then retrieved to determine whether the papers were suitable. Discrepancies were settled via consensus.

### Data extraction and quality assessment

For each trial included, we extracted the following data: first author, publication year, study design, country, treatment regimen, sample size, age, and reported outcomes, including OS, PFS, ORR, and TRAEs. We extracted the reported outcomes of each study at the endpoint of follow-up. The Cochrane Risk of Bias tool [24] was used to assess the risk of bias in the included studies. The data extraction and the risk of bias were conducted independently by Jing Kang and Jun Zhang.

### Statistical analysis

We estimated the pooled HR and 95% CI for OS and PFS as well as the pooled OR and 95% CI for ORR and TRAEs. To investigate heterogeneity, *I*^2^ statistics were calculated. If the *I*^2^ value was more than 50%, a random-effect model was used to pool the results; otherwise, a fixed-effect model was utilized. Begg’s and Egger’s tests were used to assess the risk of publication bias across studies, with a *P* value of <0.05 indicating the existence of bias. To test the stability of the results, sensitivity analyses were performed. Stata version 12 was used for all statistical analyses.

## Results

### Search results

**Fig 1:**
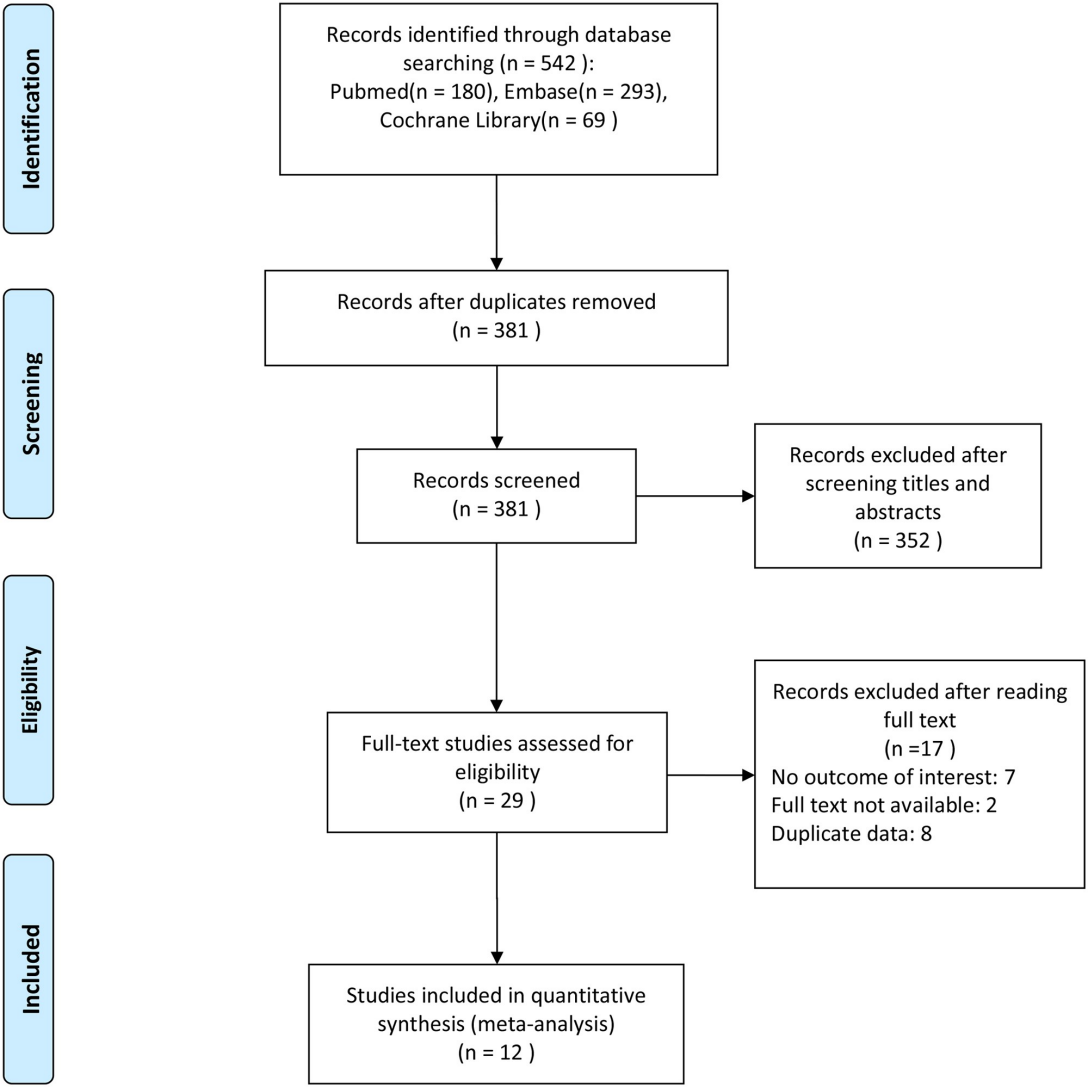
Study search and selection flow diagram.

Our literature search identified 542 articles potentially eligible for this study. After removing duplicates from the 542 articles found, 381 remained. After reviewing the titles and abstracts, 29 articles were chosen as potentially appropriate. Twelve studies were included in our meta-analysis after reviewing the abstracts and full texts. The literature search process is illustrated in Fig 1.

### Characteristics of included studies

The main characteristics of the included studies are presented in Table 1. They are all RCTs published between 2012 and 2021. Among the 12 RCTs, four trials investigated PD-L1 inhibitors; six trials investigated PD-1 inhibitors; and two trials investigated CTLA-4 inhibitors. Two trials investigated ICIs plus targeted therapy plus chemotherapy, and another ten investigated ICIs plus chemotherapy. All of the patients had advanced or metastatic NSCLC (stage IIIB to IV or recurrent). Patients with nonsquamous NSCLC were enrolled in seven studies. Additionally, patients with squamous NSCLC were recruited in three trials, and patients with squamous or nonsquamous NSCLC were enrolled in two trials.

**Table 1:** Characteristics of the 12 included RCTs.

Author (year)	Study design	Histology	Stage	Intervention	Control	Sample size (I/C)	Percent female (I/C)	Mean age (year) (I/C)
Lynch et al (2012) [33]	RCT	NSCLC	IIIB/IV or recurrent	IPI+CT	CT	68/66	28/17	61/62
Govindan et al (2017) [34]	RCT	Squamous NSCLC	IV or recurrent	IPI+CT	CT	388/361	16/14	64/64
West et al (2019) [35]	RCT	Non-squamous NSCLC	IV	ATE+CT	CT	483/240	43/43	64/65
Reck et al (2019) [29]	RCT	Non-squamous NSCLC	IV	ATE+BEV+CT	BEV+CT	400/400	40/40	63/63
Jotte et al (2020) [36]	RCT	Squamous NSCLC	IV	ATE+CT	CT	343/340	18.4/18.4	65/65
Paz-Ares et al (2020) [37]	RCT	Squamous NSCLC	IV	PEM+CT	CT	278/281	20.9/16.4	65/65
Awad et al (2021) [18]	RCT	Non-squamous NSCLC	IIIB/IV	PEM+CT	CT	60/63	63/59	62.5/66.0
Zhou et al (2021) [22]	RCT	Non-squamous NSCLC	IIIB/IV	CAM+CT	CT	205/207	29/28	59/61
Nishio et al (2021) [19]	RCT	Non-squamous NSCLC	IV	ATE+CT	CT	292/286	34.2/32.9	64/63
Arrieta et al (2020) [38]	RCT	NSCLC	Metastatic	PEM+CT	CT	40/38	52/66	50.1/62.1
Rodríguez-Abreu et al (2021) [20]	RCT	Non-squamous NSCLC	IV	PEM+CT	CT	410/206	38/47.1	65/63.5
Sugawara et al (2021) [21]	RCT	Non-squamous NSCLC	IIIB/IV or recurrent	NIV+BEV+CT	BEV+CT	275/275	25.5/25.1	66/66

RCTs: randomized controlled trials; I: intervention; C: control; NSCLC: non-small cell lung cancer; IPI: ipilimumab; ATE: atezolizumab; PEM: pembrolizumab; CAM: camrelizumab; NIV: nivolumab; BEV: bevacizumab; CT: chemotherapy.

### Quality of studies in the analysis

We divided the studies into three categories as follows: low, uncertain, and high risk of bias. Three studies had a low risk of bias; two had a risk of bias that was uncertain; and seven had a high risk of bias (S2 Table). Some studies did not provide enough information on sequence generation or allocation concealment, or they reported insufficient results.

#### Efficacy

**Fig 2:**
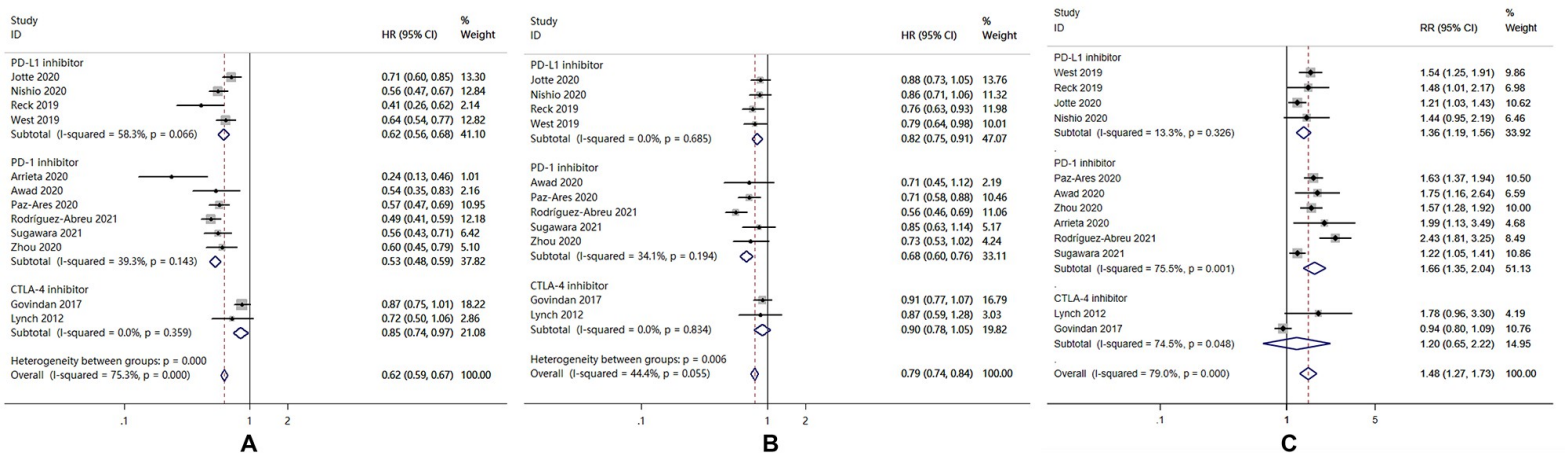
Forest plot of meta-analysis for hazard ratios comparing (A) OS, (B) PFS, and (C) ORR in patients who received ICIs plus chemotherapy versus chemotherapy.

This meta-analysis included 11 studies for OS, 12 studies for PFS, and 12 studies for ORR. Overall, the analysis indicated that ICIs plus chemotherapy could significantly improve OS (HR = 0.79; 95% CI: 0.74–0.84; *I*^2^ = 44.4%, *P* = 0.055) (Fig 2A), PFS (HR = 0.62; 95% CI: 0.59–0.67; *I*^2^ = 75.3%, *P* = 0.000) (Fig 2B), and ORR (RR = 1.48; 95% CI: 1.27–1.73; *I*^2^ = 79.0%, *P* = 0.000) (Fig 2C) compared to chemotherapy treatments.

Subgroup analysis revealed that PD-L1 inhibitors combined with chemotherapy and PD-1 inhibitors combined with chemotherapy were associated with improved OS (HR = 0.82, 95% CI: 0.75–0.91; HR = 0.68, 95% CI: 0.60–0.76, respectively) (Fig 2A); PFS (HR = 0.62, 95% CI: 0.56–0.68; HR = 0.53, 95% CI: 0.48–0.59, respectively) (Fig 2B), and ORR (RR = 1.36, 95% CI: 1.19–1.56; RR = 1.66, 95% CI: 1.35–2.04, respectively) (Fig 2C). However, CTLA-4 inhibitors combined with chemotherapy only improved PFS (HR = 0.85, 95% CI: 0.74–0.97) (Fig 2B), but did not improve OS (HR = 0.90; 95% CI: 0.78–1.05) (Fig 2A) or ORR (RR = 0.99; 95% CI: 0.85–1.15) (Fig 2C).

#### Safety

**Fig 3:**
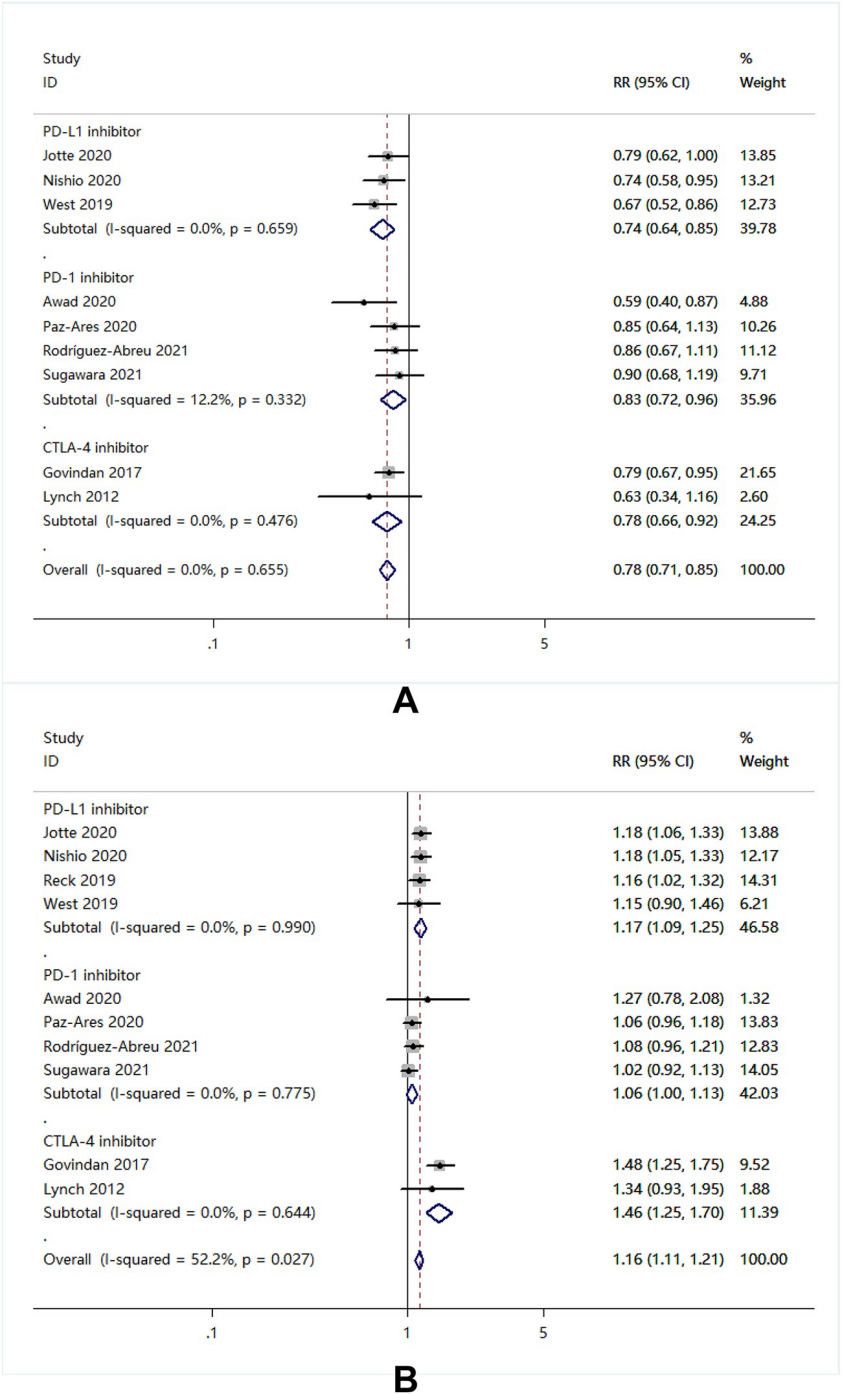
Forest plot of meta-analysis of immune-related adverse events with ICIs plus chemotherapy versus chemotherapy. (A) grade 1–2 treatment-related adverse events, (B) grade 3–5 treatment-related adverse event.

Compared with chemotherapy alone, the combination of chemotherapy decreased the risk of grade 1–2 TRAEs with PD-L1 (RR = 0.74; 95% CI: 0.64–0.85), PD-1 (RR = 0.83; 95% CI: 0.72–0.96), and CTLA-4 (RR = 0.78; 95% CI: 0.66–0.92) (Fig 3A). Conversely, in terms of grade 3–5 TRAEs, the combination of ICIs and chemotherapy increased the risk (RR = 1.16; 95% CI: 1.11–1.21) (Fig 3B). Subgroup analysis revealed that the grade 3–5 TRAEs risk of PD-L1 inhibitors plus chemotherapy or CTLA-4 inhibitors plus chemotherapy was higher than chemotherapy as a stand-alone treatment (RR = 1.17; 95% CI: 1.09–1.25), (RR = 1.46; 95% CI: 1.25–1.70). However, the risk of grade 3–5 TRAEs did not increase in PD-1 inhibitors plus chemotherapy (RR = 1.06; 95% CI: 1.00–1.13) (Fig 3B).

### Subgroup analysis

We conducted subgroup analyses based on histological type, age, sex, smoker status, liver metastasis, PD-L1 expression, and pathology. Female patients (female, HR = 0.59, 95% CI: 0.49–0.70 vs. male HR = 0.87, 95% CI: 0.77–0.98) might receive more OS benefit from ICIs plus chemotherapy. Patients with nonsquamous histology (nonsquamous HR, 0.56, 95% CI: 0.51–0.61 vs. squamous HR = 0.73, 95% CI: 0.66–0.81) or with high PD-L1 expression (high PD-L1 expression HR = 0.42, 95% CI: 0.33–0.53 vs. low PD-L1 expression HR = 0.65, 95% CI: 0.56–0.75) might receive more PFS benefit from the combination therapy. However, other factors failed to predict OS or PFS benefit with ICIs plus chemotherapy (Table 2). PD-1/PD-L1 inhibitors plus chemotherapy improved ORRs across all tested subgroups of PD-L1 expression level, including those with a PD-L1 high expression level (HR, 1.51; 95% CI: 1.21–1.87), PD-L1 low expression level (HR, 1.26; 95% CI: 1.06–1.48), and PD-L1 negative (HR, 1.35; 95% CI, 1.20–1.51) (Table 3).

**Table 2:** Subgroup analysis of OS, PFS of ICIs plus chemotherapy vs chemotherapy in NSCLC.

Variable	No of studies	HR with 95% CI	Heterogeneity		Study	No of studies	HR with 95% CI	Heterogeneity	
			*I*^2^(%)	*P* value				*I*^2^(%)	*P* value
**OS**					**PFS**				
Overall	11	0.79 (0.74,0.84)	44.4	0.055	Overall	12	0.62 (0.59,0.67)	75.3	0.000
Histological type					Histological type				
Squamous	3	0.84 (0.76, 0.940)	44.1	0.097	Squamous	3	0.73 (0.66,0.81)	83.1	0.003
Non-squamous	7	0.74 (0.68,0.81)	45.2	0.161	Non-squamous	7	0.56 (0.51,0.61)	7.2	0.373
Sex					Sex				
Male	4	0.87 (0.77,0.98)	0.0	0.599	Male	5	0.63 (0.57,0.70)	4.3	
Female	4	0.59 (0.49,0.70)	62.6	0.045	Female	6	0.53 (0.47,0.61)	38.6	0.149
Age					Age				
>65 year	4	0.79 (0.69,0.92)	0.0	0.867	>65 year	6	0.62 (0.55,0.70)	0.0	0.881
<65 year	4	0.75 (0.65,0.86)	74.6	0.008	<65 year	6	0.59 (0.53,0.66)	58.8	0.033
Smoker					Smoker				
Never	4	0.56 (0.41,0.78)	54.6	0.085	Never	5	0.59 (0.46,0.76)	0.0	0.672
Ever	4	0.80 (0.72,0.88)	58.0	0.068	Ever	5	0.60 (0.55,0.65)	47.0	0.110
Liver metastasis					Liver metastasis				
Yes	5	0.83 (0.68,1.01)	53.8	0.07	Yes	6	0.67 (0.56,0.80)	41.8	0.126
No	4	0.74 (0.66,0.83)	56.9	0.07	No	4	0.60 (0.57,0.62)	49.0	0.117
PD-L1 expression					PD-L1 expression				
PD-L1 high	4	0.69 (0.52,0.92)	0.0	0.646	PD-L1 high	4	0.42 (0.33,0.53)	0.0	0.532
PD-L1 low	4	0.93 (0.78,1.12)	43.8	0.149	PD-L1 low	4	0.65 (0.56,0.75)	3.1	0.377
PD-L1 positive	4	0.72 (0.626,0.84)	13.0	0.328	PD-L1 positive	5	0.52 (0.46,0.59)	35.7	0.183
PD-L1 negative	4	0.75 (0.67,0.85)	27.4	0.219	PD-L1 negative	9	0.68 (0.62,0.76)	39.2	0.107

ICIs: Immune-checkpoint inhibitors; NSCLC: non-small cell lung cancer; OS: overall survival; PFS: progression-free survival.

**Table 3:** Subgroup analysis of ORR of ICIs plus chemotherapy in NSCLC.

Variable	No of studies	RR with 95% CI	Heterogeneity		Study	No of studies	RR with 95% CI	Heterogeneity	
			*I*^2^(%)	*P* value				*I*^2^(%)	*P* value
**PD-L1 high**	3	1.51 (1.21,1.87)	0.0	0.438	**PD-L1 positive**	4	1.64 (1.44,1.88)	68.3	0.024
PD-L1 low	3	1.26 (1.06,1.48)	13.7	0.314	PD-L1 negative	7	1.35 (1.20,1.51)	68.2	0.004

ICIs: Immune-checkpoint inhibitors; NSCLC: non-small cell lung cancer; ORR: objective response rate.

### Sensitivity analysis

Because there was significant heterogeneity in PFS, ORR, and TRAEs, we assessed the influence of each study on the pooled results. The results revealed that the outcomes of OS, PFS, ORR, grade 1–2 TRAEs, and grade 3–5 TRAEs were reliable and stable (S1–S5 Figs).

### Publication bias

Egger’s and Begg’s tests were used to evaluate the publication bias of the OS, PFS, ORR, and TRAEs. No evidence of publication bias was found for OS, PFS, ORR, or grade 3–5 TRAEs (S6–S10 Figs).

## Discussion

The pooled analysis, including 12 RCTs of high quality involving 6005 patients, found that PD-1/PD-L1 inhibitors combined with chemotherapy significantly improved OS, PFS, and ORR when compared to chemotherapy with reduced grade 1–2 TRAEs. Moreover, female patients with nonsquamous histology might receive more OS benefit from ICIs plus chemotherapy when compared to chemotherapy alone. Despite the fact that CTLA-4 inhibitors in combination with chemotherapy increased PFS, there were no improvements in terms of OS or ORR. The addition of PD-L1/CTLA-4 inhibitors to chemotherapy was associated with an increased risk of grade 3–5 TRAEs while PD-1 inhibitors were not.

In terms of ORR, PFS, and OS, our meta-analysis found some differences between PD-1 and PD-L1 inhibitors with chemotherapy for patients with NSCLC. Another meta-analysis found that anti–PD-1 was more effective than anti–PD-L1 as monotherapy in patients with metastatic and previously treated NSCLC [25] or as a first-line treatment for advanced squamous NSCLC combined with chemotherapy [26]. PD-1 inhibitors plus chemotherapy, as first-line treatment for NSCLC patients, demand further study. In my systematic review, grades 3–5 TRAEs were more prevalent with CTLA-4 inhibitors combined with chemotherapy than with PD-1 inhibitors combined with chemotherapy. Similarly, one systematic review showed that grade 3–4 immune-related adverse events (irAEs) were more prevalent with CTLA-4 inhibitors than with PD-1 inhibitors (31% versus 10%) for cancer treatment [27]. Compared to PD-1/PD-L1 inhibitors, CTLA-4 inhibitors were weak in treating small cell lung cancer with high side effects, which may be the reason why there were fewer RCTs of CTLA inhibitors for small cell lung cancer. For patients with advanced nonsquamous NSCLC, a combination of platinum-based chemotherapy with bevacizumab, a monoclonal antibody against vascular endothelial growth factor (VEGF), resulted in modest improvements in survival [28]. In treatment of NSCLC, adding PD-1/PD-L1 inhibitors to platinum-based chemotherapy and bevacizumab seems to increase therapeutic efficacy. However, when PD-L1 inhibitors were added to platinum-based chemotherapy and bevacizumab, the incidence of grade 3–5 adverse events rose although PD-1 inhibitors did not [21, 29]. Therefore, combination therapy with PD-1 inhibitors showed increased clinical benefit for patients with NSCLC. In the future, additional basic research on targeted medicines and ICIs may be required to produce more effective therapeutic agents that can be used in combination with chemotherapy.

In the subgroup analysis, ICIs combined with chemotherapy were likely to improve OS in female patients and PFS in PD-L1-positive patients. Our study also revealed a significant improvement of PFS in nonsquamous NSCLC (nonsquamous HR, 0.56 vs. squamous HR, 0.73). This appears to support the evidence that people with nonsquamous NSCLC benefit more from checkpoint inhibitors [30]. This meta-analysis further revealed that age was not predictive of OS benefit with ICIs combined with chemotherapy versus chemotherapy. This finding is different from previous meta-analyses that revealed greater efficacy with immune checkpoint inhibitor monotherapy in patients older than 65 years old [14]. Furthermore, when compared to chemotherapy, single-agent ICIs revealed significant progression-free survival (PFS) improvements in nonsmokers, men, and PD-L1- positive patients in a meta-analysis [31]. This suggests that the addition of chemotherapy to ICIs may alter treatment outcomes for certain subtypes, such as smoking history, age, and liver metastases compared to immunosuppressants. Such results can provide many clinical implications: for females with high PD-L1 expression, it is preferable to choose ICIs combined with chemotherapy.

The high quality of evidence provided in the meta-analysis is one of the work’s strengths. The data for this study came from 12 updated RCTs with a total of 6005 individuals. In addition, we performed subgroup analyses according to histological type, age, sex, smoker status, liver metastasis, PD-L1 expression, and pathology, which will provide some guidance for the treatment of different subgroups of NSCLC. The included literature of the previous two meta-analyses was published before 2020 [14, 17], and many recently published articles were not included, but our meta-analysis incorporates the most recently updated RCTs as well as novel recently published RCTs. In addition, they did not discuss the side effects of PD-1 inhibitors plus chemotherapy and PD-L1 inhibitors plus chemotherapy separately; however, when PD-L1/CTLA-4 inhibitors were added to chemotherapy, the risk of grade 3–5 adverse events increased, whereas PD-1inhibitors were not included in our meta-analysis. Another meta-analysis suggested that chemotherapy plus ICIs might improve PFS and ORR compared with single-agent ICIs for advanced NSCLC patients with PD-L1 ≥ 50%. However, it did not lead to an OS benefit. However, our meta-analysis found that ICIs plus chemotherapy could significantly improve OS. The difference in results may be caused by concerns about advanced NSCLC patients with PD-L1 ≥ 50% being different from ours. Another meta-analysis focused only on stage 3 non-small cell carcinoma [32] while our advanced NSCLC encompassed both stages 3 and 4. Our meta-analysis, however, has certain limitations. First, we did not stratify the analysis of particular ICIs in our meta-analysis. This may account for some of the results’ heterogeneity, and the pooled results may differ somewhat from the effects of specific ICIs. Second, we may have overlooked trials that were not written in English or were unpublished leading to an overestimation of the efficacy of these therapies. Third, our analysis included two trials employing CTLA-4 inhibitors, which may have led to reporting bias.

## Conclusions

ICIs plus chemotherapy, compared with chemotherapy, were associated with significantly improved PFS, ORR, and OS in NSCLC therapy. However, PD-L1/CTLA-4 inhibitors plus chemotherapy could increase the risk of grade 3–5 adverse events, but not PD-1 inhibitors plus chemotherapy.

## Supporting information

S1 Checklist PRISMA 2020 checklist. (DOCX)

S1 Table Summary of search strategies. (DOCX)

S2 Table Risk of bias of randomized controlled trials included in this meta-analysis. (DOCX)

S1 Fig Sensitivity analysis for the overall survival meta-analysis. (TIF)

S2 Fig Sensitivity analysis for progression-free survival meta-analysis. (TIF)

S3 Fig Sensitivity analysis for objective response rate meta-analysis. (TIF)

S4 Fig Sensitivity analysis for grade 1–2 adverse events meta-analysis. (TIF)

S5 Fig Sensitivity analysis for grade 3–5 adverse events meta-analysis. (TIF)

S6 Fig The results of the Begg’s and Egger’s test for studies that reported overall survival. (PNG)

S7 Fig The results of the Begg’s and Egger’s test for studies that reported progression-free survival. (PNG)

S8 Fig The results of the Begg’s and Egger’s test for studies that reported objective response rate. (PNG)

S9 Fig The results of the Begg’s and Egger’s test for studies that reported grade 1–2 treatment-related adverse events. (PNG)

S10 Fig The results of the Begg’s and Egger’s test for studies that reported grade 3–5 treatment-related adverse events. (PNG)
