# Supplementary material for: The efficacy and safety of immune-checkpoint inhibitors plus chemotherapy versus chemotherapy for non-small cell lung cancer: An updated systematic review and meta-analysis
Source: PLoS One. 2024 Feb 6;19(2):e0276318. doi: 10.1371/journal.pone.0276318 (PMC10846740; doi:10.1371/journal.pone.0276318)
Supplement: S1 Table — (DOCX) [file pone.0276318.s002.docx]

| **S1 Table. Summary of search strategies** | | |
| --- | --- | --- |
| **Pubmed** | | |
| Search | Query | Results |
| #1 | (((((((((((("Carcinoma, Non-Small-Cell Lung"[Mesh]) OR (Carcinoma, Non Small Cell Lung[Title/Abstract])) OR (Carcinomas, Non-Small-Cell Lung[Title/Abstract])) OR (Lung Carcinoma, Non-Small-Cell[Title/Abstract])) OR (Lung Carcinomas, Non-Small-Cell[Title/Abstract])) OR (Non-Small-Cell Lung Carcinomas[Title/Abstract])) OR (Non-Small-Cell Lung Carcinomas[Title/Abstract])) OR (Non Small Cell Lung Carcinoma[Title/Abstract])) OR (Carcinoma, Non-Small Cell Lung[Title/Abstract])) OR (Non-Small Cell Lung Carcinoma[Title/Abstract])) OR (Non-Small Cell Lung Cancer[Title/Abstract])) OR (Nonsmall Cell Lung Cancer[Title/Abstract])) | 64245 |
| #2 | ((((((((((((((immune therapy[Title/Abstract]) OR (immunotherapy[Title/Abstract])) OR (immune checkpoint blockade[Title/Abstract])) OR (immune checkpoint inhibitor[Title/Abstract])) OR (PD-1[Title/Abstract])) OR (PD-L1[Title/Abstract])) OR ( CTLA-4[Title/Abstract])) OR (durvalumab[Title/Abstract])) OR (avelumab[Title/Abstract])) OR (tremelimumab[Title/Abstract])) OR (atezolizumab[Title/Abstract])) OR (nivolumab[Title/Abstract])) OR (pembrolizumab[Title/Abstract])) OR (ipilimumab[Title/Abstract])) OR (immune vaccine[Title/Abstract]) | 119346 |
| #3 | #1 AND #2 | 5120 |
| #4 | #3 AND (Randomized Controlled Trial [Filter]) | 180 |
| **Embase** | | |
| #1 | 'non small cell lung cancer'/exp OR 'carcinoma, non-small-cell lung':ab,ti OR 'carcinoma, non small cell lung':ab,ti OR 'carcinomas, non-small-cell lung':ab,ti OR 'lung carcinoma, non-small-cell':ab,ti OR 'lung carcinomas, non-small-cell':ab,ti OR 'non-small-cell lung carcinomas':ab,ti OR 'non small cell lung carcinoma':ab,ti OR 'carcinoma, non-small cell lung':ab,ti OR 'non-small cell lung carcinoma':ab,ti OR 'non-small cell lung cancer':ab,ti | 190558 |
| #2 | 'immunotherapy'/exp OR 'immune therapy':ab,ti OR 'immune checkpoint blockade':ab,ti OR 'immune checkpoint inhibitor':ab,ti OR 'pd 1':ab,ti OR 'pd l1':ab,ti OR 'ctla 4':ab,ti OR durvalumab:ab,ti OR avelumab:ab,ti OR tremelimumab:ab,ti OR atezolizumab:ab,ti OR nivolumab:ab,ti OR pembrolizumab:ab,ti OR ipilimumab:ab,ti OR 'immune vaccine':ab,ti | 317430 |
| #3 | #1 AND #2 | 695 |
| #4 | #1 AND #2 AND [humans]/lim AND [english]/lim AND [clinical study]/lim AND [randomized controlled trial]/lim AND [article]/lim | 293 |
| **Cochrane Library** | | |
| #1 | MeSH descriptor: [Carcinoma, Non-Small-Cell Lung] this term only | 4647 |
| #2 | (Carcinoma, Non Small Cell Lung):ti,ab,kw OR (Carcinomas, Non-Small-Cell Lung):ti,ab,kw OR (Lung Carcinoma, Non-Small-Cell):ti,ab,kw OR (Lung Carcinomas, Non-Small-Cell):ti,ab,kw OR (Non-Small-Cell Lung Carcinomas):ti,ab,kw OR (Non-Small-Cell Lung Carcinomas):ti,ab,kw OR (Non Small Cell Lung Carcinoma):ti,ab,kw OR (Carcinoma, Non-Small Cell Lung):ti,ab,kw OR (Non-Small Cell Lung Carcinoma):ti,ab,kw OR (Non-Small Cell Lung Cancer):ti,ab,kw | 14434 |
| #3 | #1 AND #2 | 37828 |
| #4 | MeSH descriptor: [Immunotherapy] this term only | 1236 |
| #5 | (immune therapy):ti,ab,kw OR (immune checkpoint blockade):ti,ab,kw OR (immune checkpoint inhibitor):ti,ab,kw OR (PD-1):ti,ab,kw OR (PD-L1):ti,ab,kw OR (CTLA-4):ti,ab,kw OR (durvalumab):ti,ab,kw OR (avelumab):ti,ab,kw OR (tremelimumab):ti,ab,kw OR (atezolizumab):ti,ab,kw OR (nivolumab):ti,ab,kw OR (pembrolizumab):ti,ab,kw OR (ipilimumab):ti,ab,kw | 24178 |
| #6 | #4 AND #5 | 25132 |
| #7 | MeSH descriptor: [Drug Therapy] this term only | 351 |
| #8 | (platinum):ti,ab,kw OR (paclitaxel):ti,ab,kw OR (carboplatin):ti,ab,kw OR (pemetrexed):ti,ab,kw | 20871 |
| #9 | #7 AND #8 | 11097 |
| #10 | (randomized controlled trial):pt OR (randomized):ti,ab,kw OR (placebo):ti,ab,kw OR (RCT):ti,ab,kw | 1314767 |
| #11 | #3 AND #6 AND #9 AND #10 | 69 |
