# Supplementary material for: The efficacy and safety of immune-checkpoint inhibitors plus chemotherapy versus chemotherapy for non-small cell lung cancer: An updated systematic review and meta-analysis
Source: PLoS One. 2024 Feb 6;19(2):e0276318. doi: 10.1371/journal.pone.0276318 (PMC10846740; doi:10.1371/journal.pone.0276318)
Supplement: S2 Table — (DOCX) [file pone.0276318.s003.docx]

| **S2 Table.** **Risk of bias of randomized controlled trials included in this meta-analysis** | | | | | | | | | |
| --- | --- | --- | --- | --- | --- | --- | --- | --- | --- |
| **Study, year** | **Sequence**  **generation** | **Allocation**  **concealment** | **Blinding of**  **participants** | **Blinding of**  **personnel** | **Blinding of**  **outcome**  **assessors** | **Incomplete**  **outcome**  **data** | **Selective**  **outcome**  **reporting** | **Other**  **sources**  **of bias** | **Summary assessments of the risk of bias** |
| **PD-1 + CT vs. CT** |  |  |  |  |  |  |  |  |  |
| Arrieta （2020） | Unclear risk | High risk | High risk | High risk | Low risk | Low risk | Low risk | Low risk | High risk |
| Awad (2020) | Low risk | High risk | High risk | High risk | Low risk | Low risk | Low risk | Low risk | High risk |
| Paz-Ares (2020) | Low risk | Low risk | Low risk | Low risk | Low risk | Low risk | Low risk | Low risk | Low risk |
| Zhou (2020) | Low risk | High risk | High risk | High risk | Low risk | Low risk | Low risk | Unclear risk | High risk |
| Rodríguez-Abreu (2021) | Unclear risk | Unclear risk | Unclear risk | Unclear risk | Low risk | Low risk | Low risk | Low risk | Unclear risk |
| Sugawara (2021) | Low risk | Low risk | Low risk | Low risk | Low risk | Low risk | Low risk | Low risk | Low risk |
| **PD-L1 + CT vs. CT** |  |  |  |  |  |  |  |  |  |
| Reck (2019) | Low risk | High risk | High risk | High risk | Unclear risk | Low risk | Low risk | Low risk | High risk |
| West (2019) | Low risk | High risk | High risk | High risk | Low risk | Low risk | Low risk | Unclear risk | High risk |
| Jotte (2020) | Unclear risk | High risk | High risk | High risk | Low risk | Low risk | Low risk | Low risk | High risk |
| Nishio (2020) | Unclear risk | High risk | High risk | High risk | Low risk | Low risk | Low risk | Unclear risk | High risk |
| **CTLA-4 + CT vs. CT** |  |  |  |  |  |  |  |  |  |
| Lynch (2012) | Unclear risk | Low risk | Low risk | Low risk | Low risk | Low risk | Low risk | Low risk | Unclear risk |
| Govindan (2017) | Low risk | Low risk | Low risk | Low risk | Low risk | Low risk | Low risk | Low risk | Low risk |
| CT: chemotherapy |  |  |  |  |  |  |  |  |  |
