# Supplementary figures and images for: The efficacy and safety of immune-checkpoint inhibitors plus chemotherapy versus chemotherapy for non-small cell lung cancer: An updated systematic review and meta-analysis
Source: PLoS One. 2024 Feb 6;19(2):e0276318. doi: 10.1371/journal.pone.0276318 (PMC10846740; doi:10.1371/journal.pone.0276318)

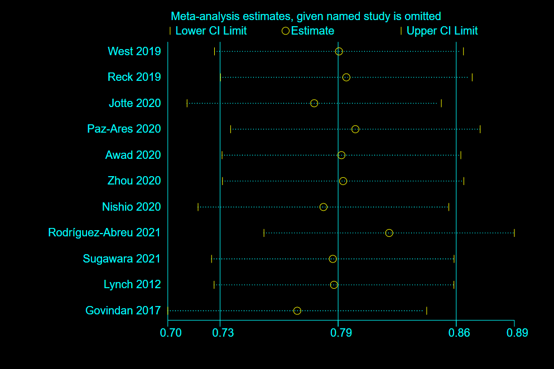

Supplement: S1 Fig — (TIF) [file pone.0276318.s004.tif]

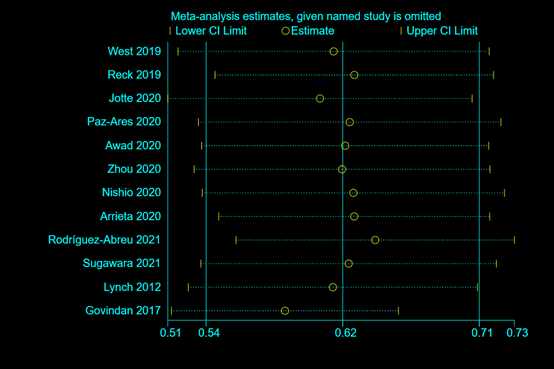

Supplement: S2 Fig — (TIF) [file pone.0276318.s005.tif]

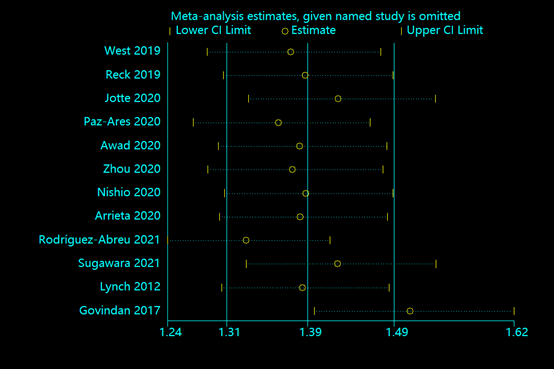

Supplement: S3 Fig — (TIF) [file pone.0276318.s006.tif]

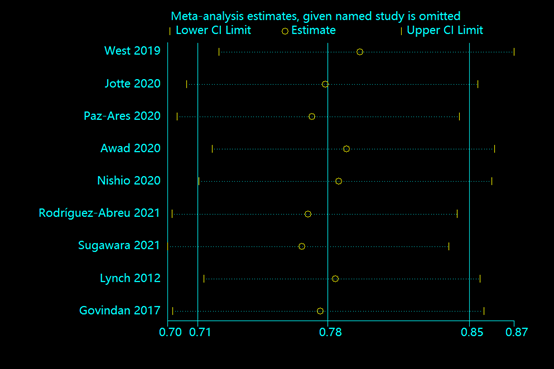

Supplement: S4 Fig — (TIF) [file pone.0276318.s007.tif]

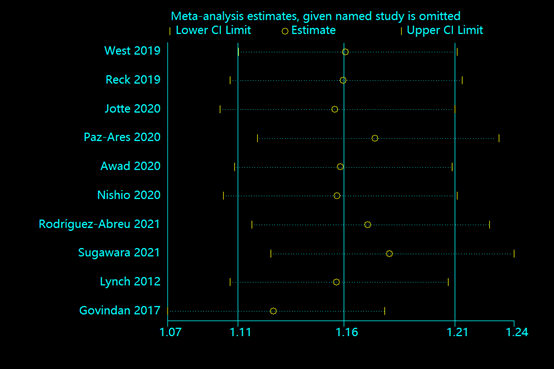

Supplement: S5 Fig — (TIF) [file pone.0276318.s008.tif]

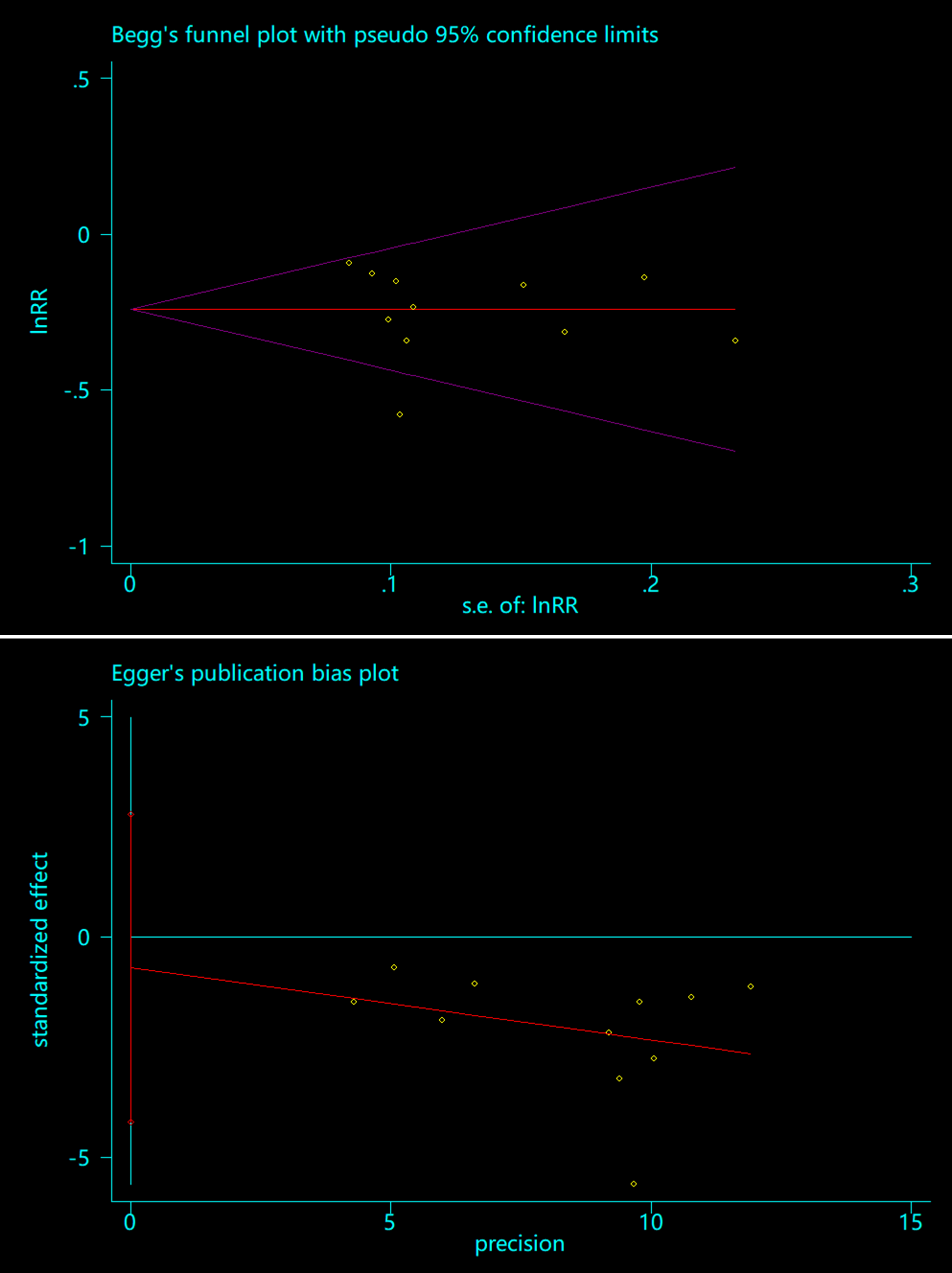

Supplement: S6 Fig — (PNG) [file pone.0276318.s009.png]

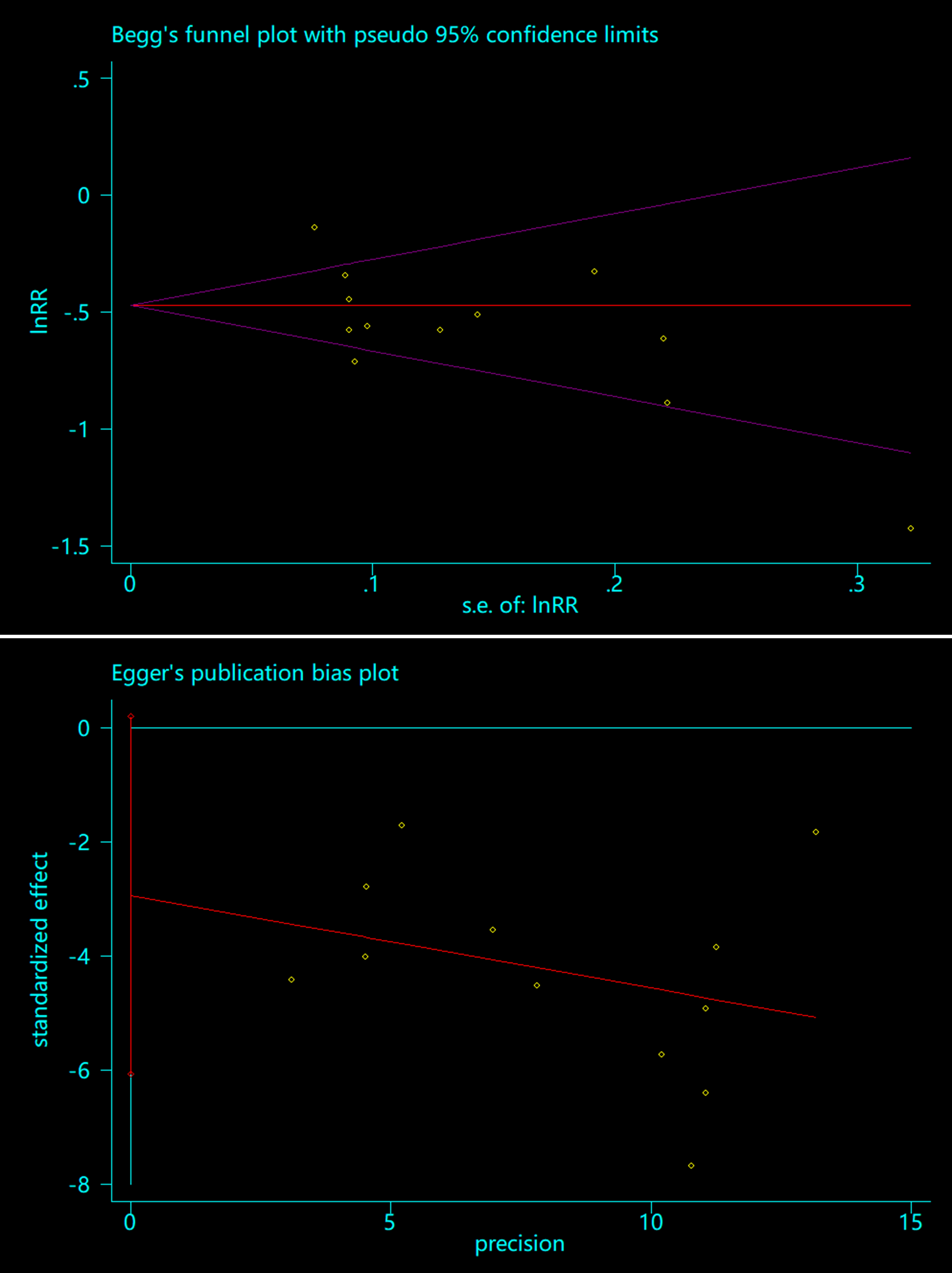

Supplement: S7 Fig — (PNG) [file pone.0276318.s010.png]

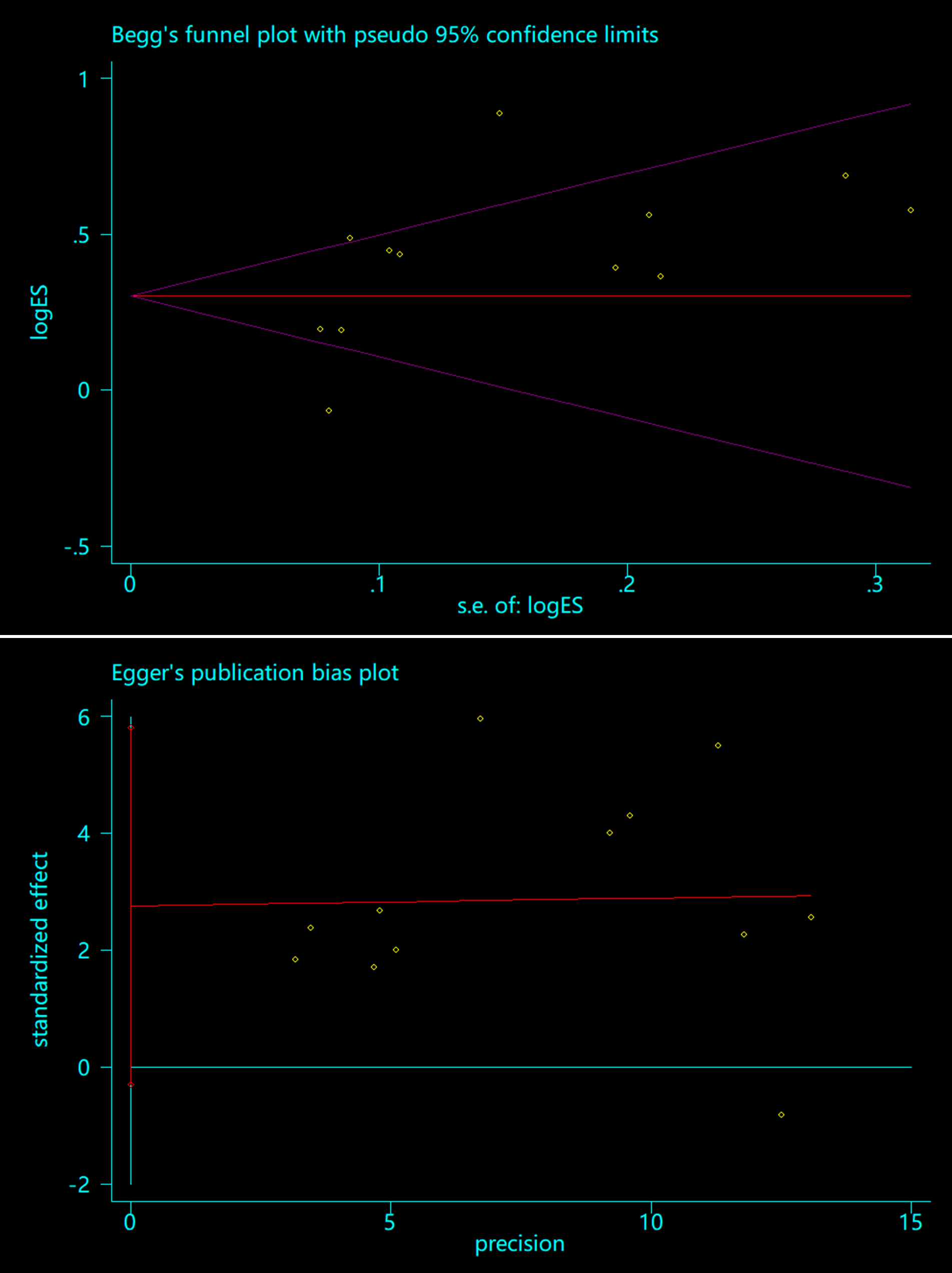

Supplement: S8 Fig — (PNG) [file pone.0276318.s011.png]

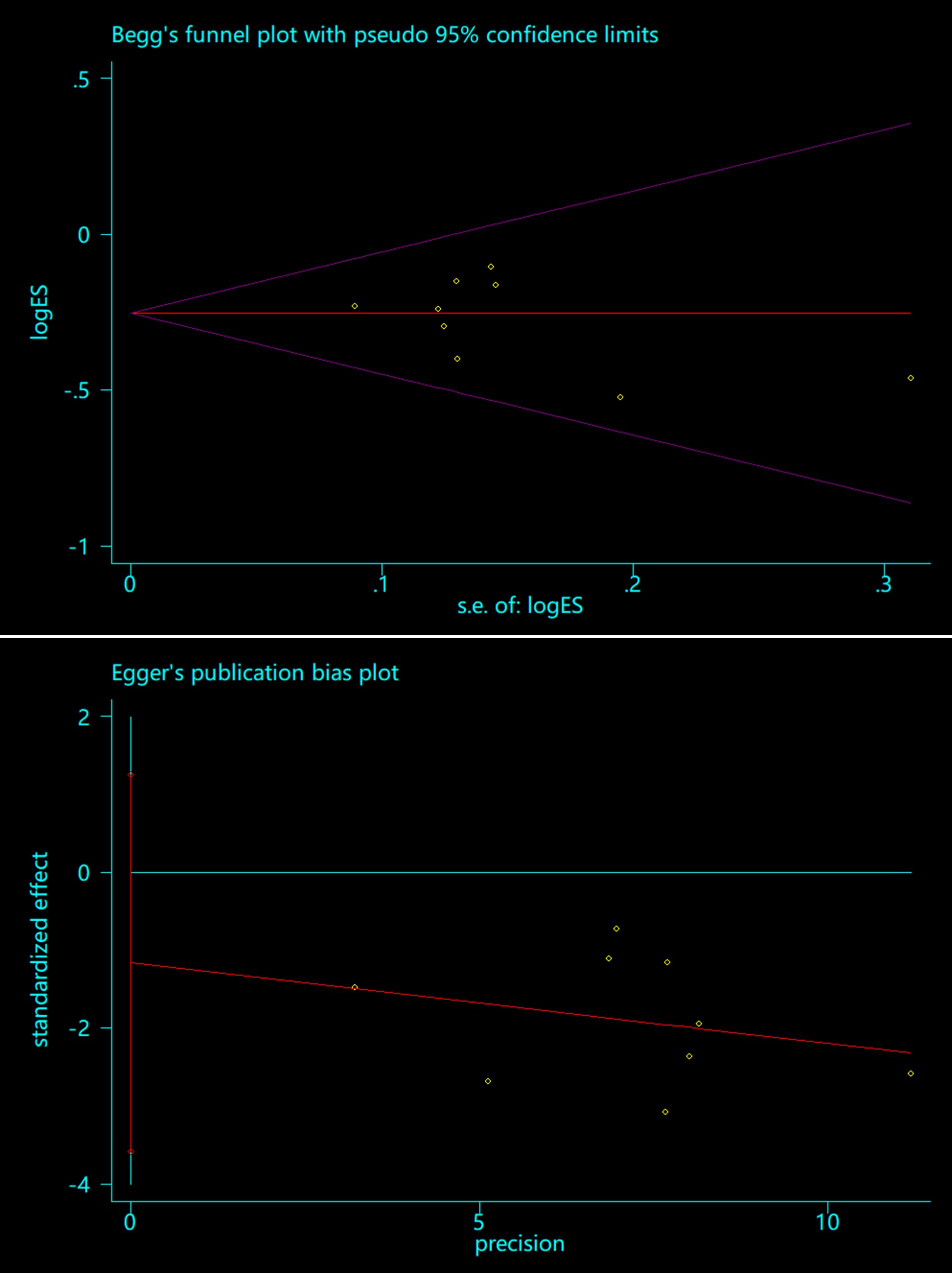

Supplement: S9 Fig — (PNG) [file pone.0276318.s012.png]

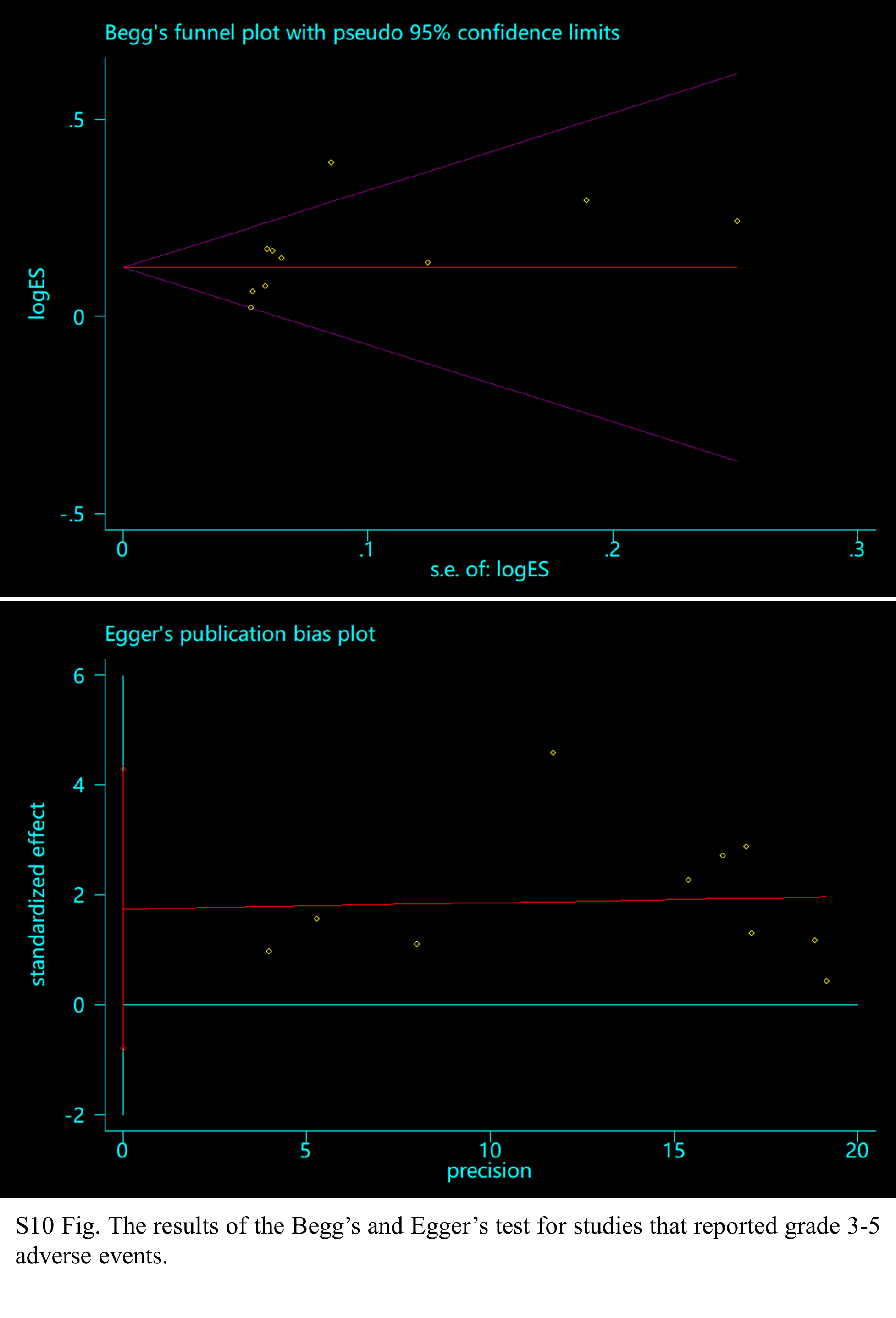

Supplement: S10 Fig — (PNG) [file pone.0276318.s013.png]
